# Live-Cell Imaging of the Association of STAT6-GFP with Mitochondria

**DOI:** 10.1371/journal.pone.0055426

**Published:** 2013-01-30

**Authors:** Rasel Khan, Jason E. Lee, Yang-Ming Yang, Feng-Xia Liang, Pravin B. Sehgal

**Affiliations:** 1 Department of Cell Biology and Anatomy, New York Medical College, Valhalla, New York, United States of America; 2 OCS Microscopy Core, New York University School of Medicine, New York, New York, United States of America; 3 Department of Medicine, New York Medical College, Valhalla, New York, United States of America; Université Joseph Fourier, France

## Abstract

The transcription factor STAT3 has been previously reported to be associated with mitochondria. However, we have been unable to visualize an association of STAT3-GFP, STAT3-DsRed or STAT3-Flag with mitochondria in human Hep3B hepatocytes thus far even though an association of these molecules with other cytoplasmic organelles (endosomes) was readily demonstrable. We then addressed the broader question of a possible association of other STAT-family of proteins with mitochondria by first using immunolocalization assays in Hep3B and human pulmonary arterial endothelial and smooth muscle cells. Strong anti-STAT6-immunolocalization with mitochondria was apparent in fluorescence and electron microscopy assays of cells first washed with a digitonin-sucrose buffer to remove bulk soluble STAT proteins. In live-cell imaging studies, STAT6-GFP, but not N1-GFP, was observed to constitutively colocalize with MitoTracker- and tetramethylrhodamine ethyl ester (TMRE)-positive mitochondria, and with mitochondrial F1-ATPase when assayed by immunofluorescence after fixation. This association was Tyr-phosphorylation independent in that a STAT6 truncated protein (STAT6^1-459^-GFP) which lacked the SH2 domain (517–632) and the cytokine-activated Y641 phosphorylation site also accumulated in MitoTracker-positive mitochondria. This was consistent with the unexpected discovery that anti-STAT6-immunofluoresence also associated with mitochondria in mouse embryo fibroblasts (MEFs) from both wild-type and the *STAT6^SH2-/SH2-^* mouse. MEFs from the latter mouse, which had been engineered in 1996 to be deleted in the STAT6 SH2 domain (amino acids 505–584) expressed an immune-specific ∼50 kDa protein detectable in whole cell and mitochondria-enriched fractions. Taken together, the present data provide the first definitive evidence of the association of any STAT-protein family member with mitochondria - that of STAT6.

## Introduction

Beginning in 2009 several investigators inferred the constitutive association of the transcription factor STAT3 with mitochondria in various human and murine cell types based upon observing the presence of STAT3 in mitochondria-enriched cell fractions as assayed by Western blotting [1], [2], [3], [4]. While, molecularly modified STAT3 carrying an engineered mitochondrial targeting sequence (MTS) was reported able to modulate mitochondrial energy-generation functions [1], [2], no microscopy evidence for the association of STAT3 with mitochondria has been forthcoming. Thus, the inference regarding mitochondrial association of STAT3 has remained controversial. Specifically, these reports [1], [3], [4] did not exclude the presence of STAT3 in association with other membranous organelles co-present in the mitochondria-enriched cell fractions. Indeed the association of STAT3 with endosomes and lysosomes had been previously characterized [5], [6], [7], [8], [9], [10]. Moreover, already in 2007 Xu et al [8] had reported that STAT3-GFP fluorescence in exogenously transfected human Hep3B hepatocytes, including that associated with IL-6-induced cytoplasmic puncta/endosomes, did not colocalize with MitoTracker-positive organelles in live-cell imaging assays in human Hep3B hepatocytes. Subsequently, Cimica et al [11] also reported that exogenously expressed STAT3-GFP did not associate with MitoTracker-positive organelles in the cytoplasm of HeLa or Hep3B cells. Additionally, Phillips et al [12] reported their inability to detect any STAT3 by mass spectrometric approaches in mitochondrial fractions derived from porcine and murine heart and liver. The absence of microscopy data (STAT3-GFP fluorescence or immunogold electron microscopy) from unfractionated cells associating STAT3 with mitochondria remained a difficulty.

The potential functional importance of the association of a STAT-protein family member with mitochondria led us to revisit the possible association of STAT3-GFP with mitochondria using a detergent-dissection approach in adherent cell cultures. In the present study a low-concentration digitonin-sucrose buffer was used to remove bulk STAT proteins from the cell cytoplasm followed by fluorescence or immunofluorescence microscopy. We remained unable to confirm the association of GFP-, DsRed- or Flag-tagged STAT3 with mitochondria. 

However, these studies led to a broader investigation of the association of other STAT family members with mitochondria. Unexpectedly strong anti-STAT6 antibody association with mitochondria was observed in human hepatocytes, endothelial and vascular smooth muscle cells in culture using immunofluorescence and immunogold electron microscopy (EM) assays. Importantly, STAT6-GFP was observed to be constitutively associated with mitochondria in live-cell assays. Moreover, we found that a 489-amino acid long N-terminal fragment of STAT6 which (a) lacked any obvious mitochondrial targeting sequence, and (b) lacked the SH2 domain and the Y641 cytokine-activated phosphorylation site was sufficient to mediate mitochondrial targeting. Additionally, we discovered that mouse embryo fibroblasts (MEFs) derived from a widely used stock of the so-called *STAT6^SH2-/SH2-^* mouse engineered to lack the SH2 domain [13], [14], [15], [16] expressed a ∼50-kDa fragment that appeared to localize to mitochondria. Overall, the present STAT6-GFP imaging data provide the first definitive evidence of the association of a STAT-protein family member with mitochondria.

## Results

### STAT3-GFP associates with cytoplasmic organelles different from mitochondria

**Figure 1:**
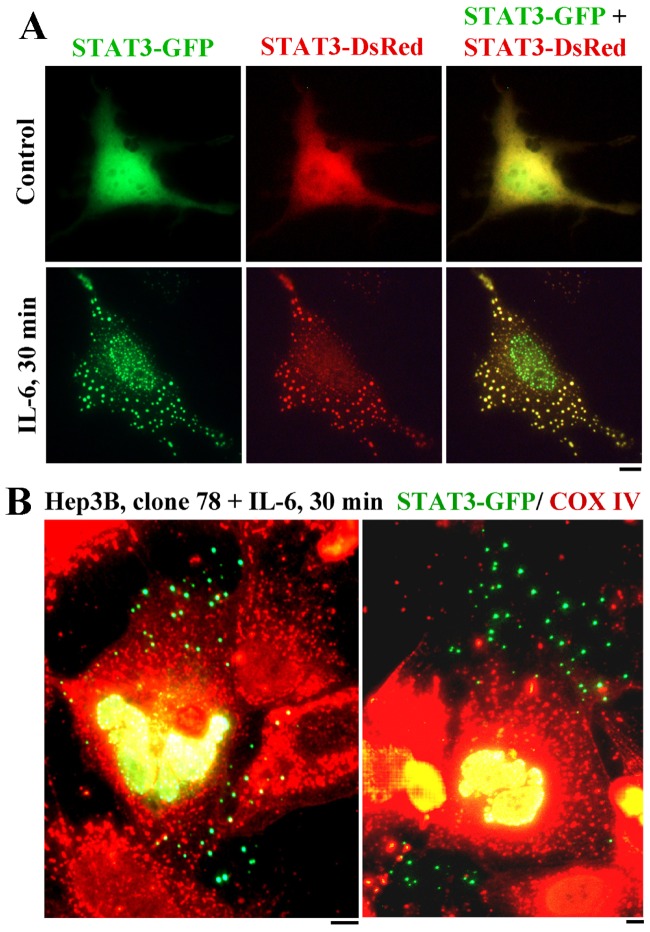
Association of STAT3-GFP with cytoplasmic sequestering endosomes in IL-6-treated Hep3B cells distinct from COX IV-positive mitochondria. Panel A. Hep3B cells in a 6-well plate were transfected with a combination of STAT3-GFP and STAT3-DsRed expression vectors and one day later imaged live without or with exposure to IL-6 for 30 min. (For convenience of subsequent imaging, at 30 min the cultures were fixed using 4% paraformaldehyde, pH 7.4, for 1 hr, washed and stored in PBS; live cell and fixed cell data were indistinguishable). Scale bar = 10 µm. Panel B. Clone 78 Hep3B cells in a 6-well plate were transfected with STAT3-GFP expression vector and one day later exposed to IL-6 for 30 min and then fixed. The cultures were then immunostained for COX IV using the rabbit pAb. Scale bar = 10 µm.

The association of fluorescently-tagged STAT3 with mitochondria was investigated in Hep3B cells under experimental conditions that have been previously shown to reveal targeting of this molecule to cytoplasmic organelles including early endosome and lysosomes [7], [8]. Interleukin-6 (IL-6) was used as the activating cytokine in the present experiments using the Hep3B hepatocyte cell line because it is well established to respond to IL-6 by increased synthesis of acute phase plasma proteins through activation of the STAT3 signaling pathway [5], [6], [7], [8]. Specifically, we have already shown that exposure of Hep3B cells to IL-6 leads to the accumulation of STAT3-GFP in cytoplasmic organelles preliminarily characterized earlier as sequestering endosomes [8]. Fig. 1A shows live-cell imaging of Hep3B cells expressing both STAT3-GFP and STAT3-DsRed before and after exposure to interleukin-6 (IL-6) for 30 min. In untreated cells, both GFP and DsRed revealed diffuse cytoplasmic fluorescence with a concentration in the cell center. After IL-6 exposure, there was the association of both the GFP and DsRed with punctate cytoplasmic organelles, as well as the apparent preferential accumulation of STAT3-GFP in puncta in the nucleus. The question of whether any of the IL-6-induced cytoplasmic STAT3-GFP structures might be mitochondrial was investigated using fixation followed by immunofluorescence for the mitochondrial protein COX IV. Fig. 1B illustrates a typical result showing the lack of such colocalization.

**Figure 2:**
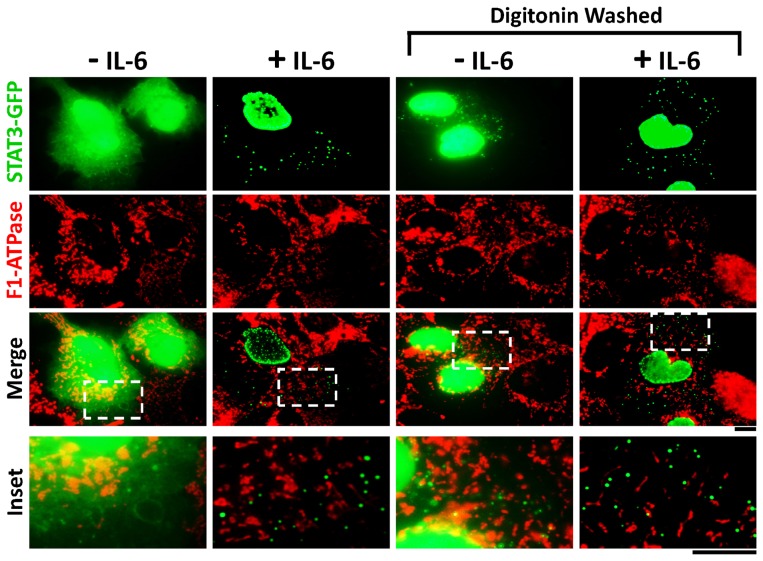
Digitonin-washed immunofluoresence assays showing lack of colocalization of STAT3-GFP with mitochondria in the cytoplasm of Hep3B cells. Hep3B cells were transfected with the STAT3-GFP expression plasmid in cultures in a 6-well plate and one day later exposed to IL-6 for 30 min or left unstimulated. The cultures were then fixed or first washed 4x with the digitonin (50 µg/ml)-0.3M sucrose buffer and then fixed followed by immunofluoresence analyses as indicated. Insets show the boxed areas (broken white lines) at higher magnification. Scale bars = 10 µm.

Since the association of STAT3 with mitochondria has been inferred to be constitutive and not cytokine-dependent [1], an alternative approach was used to try to uncover any such association. Hep3B cells expressing STAT3-GFP left untreated or exposed to IL-6 for 30 min were washed with a low-concentration digitonin-sucrose buffer followed by fixation and immunofluorescence for the mitochondrial protein F1-ATPase. Fig. 2, third column from left, shows that digitonin washing of untreated Hep3B cells removed the bulk of cytoplasmic STAT3-GFP fluorescence leaving behind punctate fluorescence in cytoplasmic organelles. The merged images in Fig. 2, bottom two rows, show that none of the digitonin-resistant STAT3-GFP-positive cytoplasmic organelles corresponded to mitochondria whether the cells had or had not been exposed to IL-6. Similar negative results were also obtained using STAT3-DsRed or STAT3-Flag (data not shown).

### Immunolocalization of STAT6 with mitochondria

**Figure 3:**
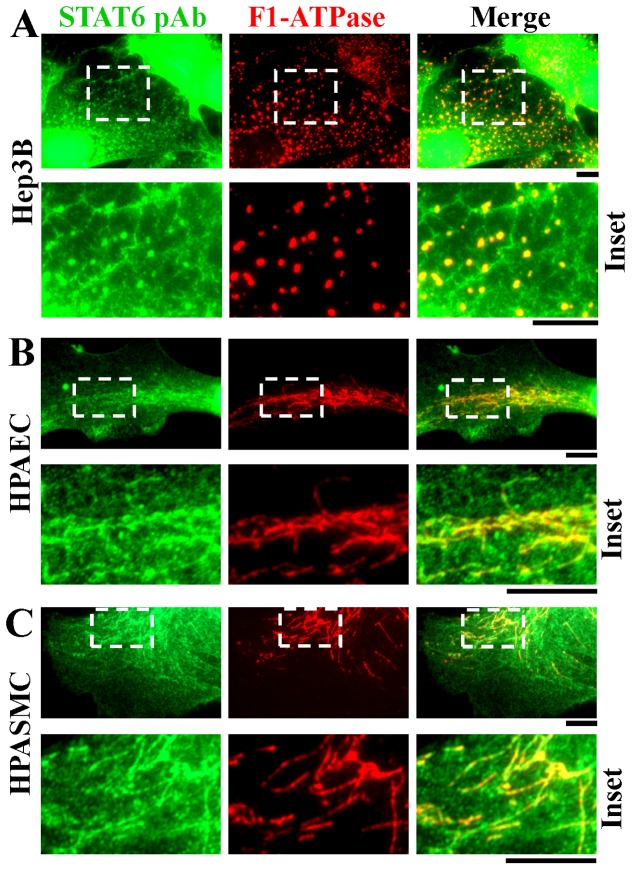
Immunoimaging assays showing colocalization of STAT6 with mitochondria in the cytoplasm of digitonin-washed Hep3B, HPAEC and HPASMC cells. The indicated cells were grown in 6-well plates for one day, washed 4x with the digitonin (50 µg/ml)-0.3M sucrose buffer and then fixed followed by immunofluoresence analyses as indicated using either the anti-STAT6 pAb (green) and F1-ATPase mAb (red). Insets are shown at high magnification within each panel. Scale bars = 10 µm. Quantitative colocalization analyses using the Pearson’s and Costes’ plugins in Image J confirmed colocalization between STAT6 and F1-ATPase immunofluorescence at a setting of *P*<0.05 in Panels A, B and C.

**Figure 4:**
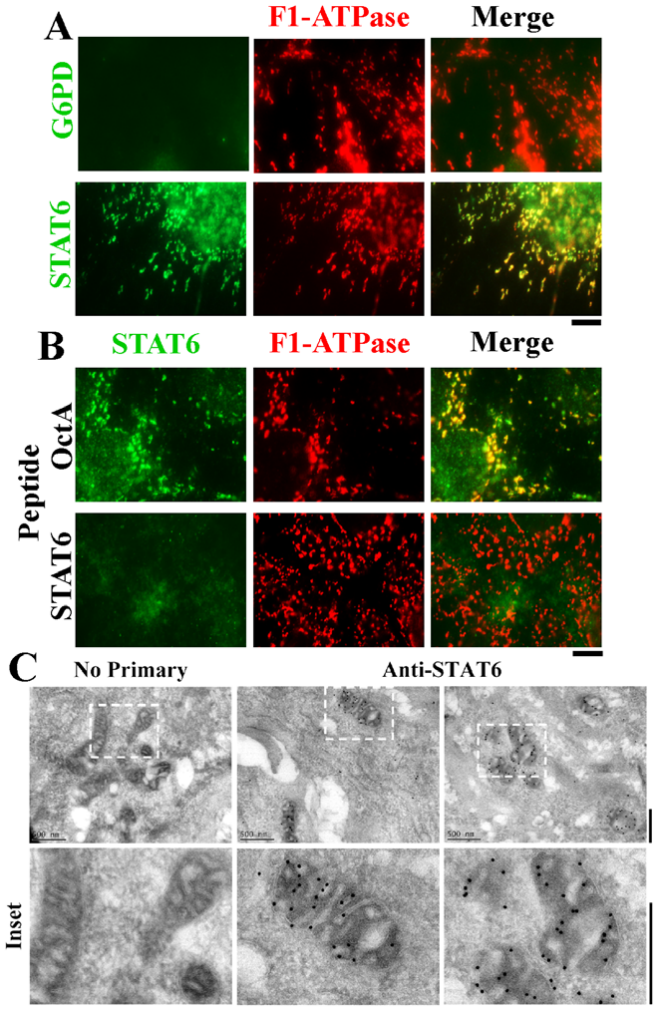
Characterization of the specificityof the anti-STAT6 immunofluoresence and its organellar localization by immunogold electron microscopy. Panel A. Anti-STAT6 immunofluoresence assayed using the rabbit pAb colocalizing with F1-ATPase in the cytoplasm of digitonin-washed Hep3B cells was compared to that of an irrelevant pAb (G6PD). Scale bar = 10 µm. Panel B. Peptide competition characterization of the anti-STAT6 pAb immunofluorescence using either the relevant STAT6 peptide that had been used as the immunogen or the irrelevant OctA peptide. Scale bar = 10 µm. Panel C. Immunogold EM localization of the anti-STAT6 pAb immunoreactivity. Cryo-thin sections of HPAECs were probed using ant-STAT6 pAb and 18-nm-tagged anti-rabbit IgG as indicated in ref. 23. Insets are shown at high magnification. Scale bars = 500 nm. Similar data were obtained using Hep3B cells (not shown).

**Figure 5:**
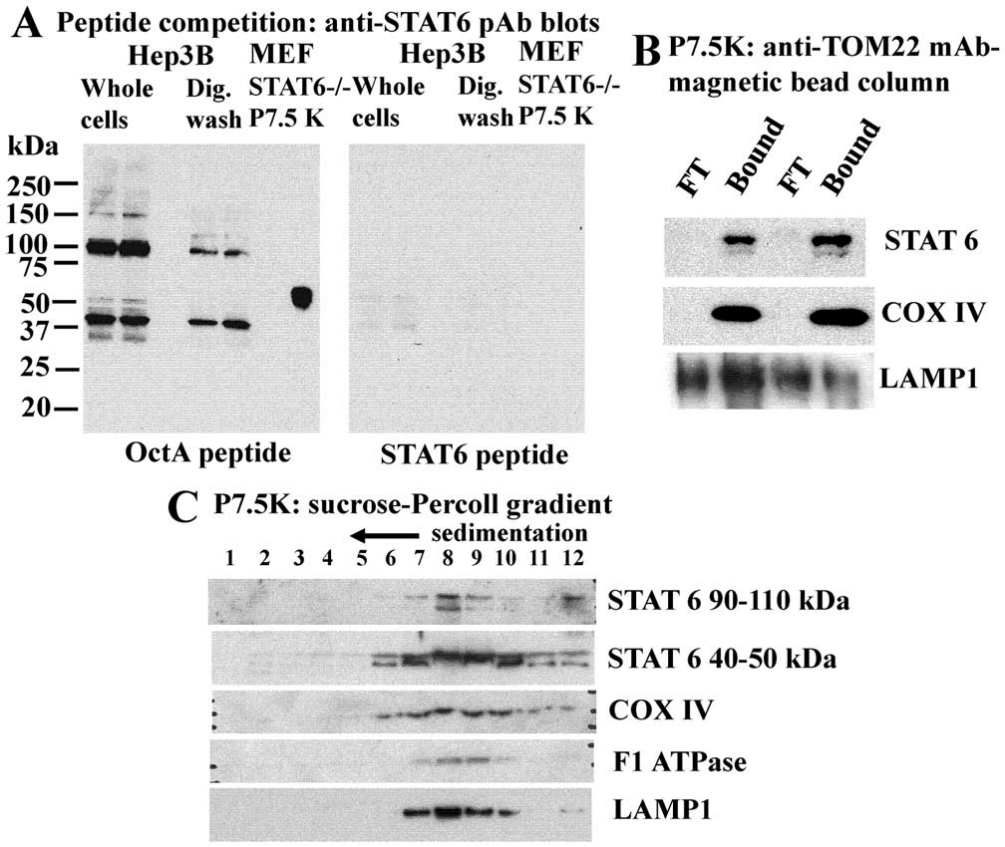
STAT6 peptides in Hep3B and *STAT6^SH2-/-SH2-^* MEF -derived cell fractions. Panel A. Western blot analyses of cell extracts (duplicate lanes) prepared from Hep3B cultures (90 mm plates) without and after washing 4x with the digitonin-sucrose buffer and the 7.5K mitochondria-enriched cell pellet derived from *STAT6^SH2-/-SH2-^* MEFs (rightmost single lane). The amount of cell extracts used in the Hep3B lanes corresponds to that derived from equal numbers of cells. Left and right blots show immunoblotting after competition with the unrelated OctA peptide or the relevant STAT6-peptide. Panel B. The 2x washed P7.5K pellet prepared from duplicate batches of Hep3B cultures (five confluent 90 mm cultures/group) were subjected to anti-TOM22-mAb magnetic bead immunoisolation chromatography. The flow-through (FT) and the Bound fractions were resedimented, checked microscopically for enrichment of mitochondria in the Bound fraction and then respective aliquots (20% of each fraction) used for Western blotting. The full-length STAT6 peptide is shown. Panel C. Hep3B cells (ten 90 mm cultures/group) were harvested and used to prepare the 2x washed P7.5K membrane pellet fraction. This was then sedimented through a Percoll (30%)-0.25 M sucrose gradient as indicated in “Materials and Methods,” the respective gradient fractions collected and prepared for Western blotting (half of each fraction for each blot) using the indicated probes.

The broader question of whether any STAT-protein family members associated with mitochondria was investigated using immunofluorescence assays and commercially available antibodies. Adherent Hep3B cells and human pulmonary arterial endothelial (HPAEC) and smooth muscle (HPASMC) cells were first washed with the digitonin-sucrose buffer to remove bulk soluble STAT proteins, followed by fixation and then Triton X-100 permeabilization and immunofluoresnece. From among all of the seven STAT-family proteins, anti-STAT6 immunofluoresence colocalizing with mitochondria in Hep3B, HPAEC and HPASMCs was the stongest (Fig. 3). This mitochondrion-associated immunofluorescence was observed using the anti-STAT6 rabbit Ab but not an unrelated rabbit Ab (to glucose 6-phosphate dehydrogenase, G6PD)(Fig. 4A), and was competed away by the relevant STAT6 immunogen peptide but not the irrelevant OctA peptide (Fig. 4B). Additionally, immunogold EM studies of HPAECs and Hep3B cells that had been first washed with the digitonin-sucrose buffer prior to fixation, cryo-embedding and thin-sectioning confirmed the localization of this anti-STAT6 immunoreactivity within mitochondria (Fig. 4C and data not shown). Western blot experiments comparing whole Hep3B cell extracts with those derived from Hep3B cultures first washed with the digitonin-sucrose buffer (as in the immunofluorescence experiments in Figs. 2 and 3) confirmed that the latter contained approximately one-fourth of the pool of full-length STAT6 as did the whole-cell extract (Fig. 5A) and that the proteins observed on Western blots were competed for by the relevant STAT6 peptide but not the irrelevant OctA peptide (Fig. 5A).

### Presence of STAT6 in mitochondria-enriched cell fractions

The association of STAT6 with isolated mitochondria-enriched cell fractions derived from Hep3B cells was investigated by preparing the traditional washed 7,500 *g* mitochondrial fraction [6], [7] followed either by further enrichment using either an anti-TOM22 mAb-magnetic bead column separation (Fig. 5B) or sedimentation through a sucrose-Percoll gradient (Fig. 5C). Both methods yielded mitochondria-enriched fractions (verified by phase microscopy and Western blotting of mitochondrial markers COX IV and F1-ATPase) that also contained STAT6 (Fig. 5B and C). However, a limitation in the interpretation of such cell-fractionation data was the detection of the lysosomal marker LAMP1 in these mitochondria-enriched fractions (Fig. 5B and C)[7]. Thus these mitochondria-enriched fractions contain other cytoplasmic membranous organelles.

### Association of STAT6-GFP with mitochondria in live-cell imaging

**Figure 6:**
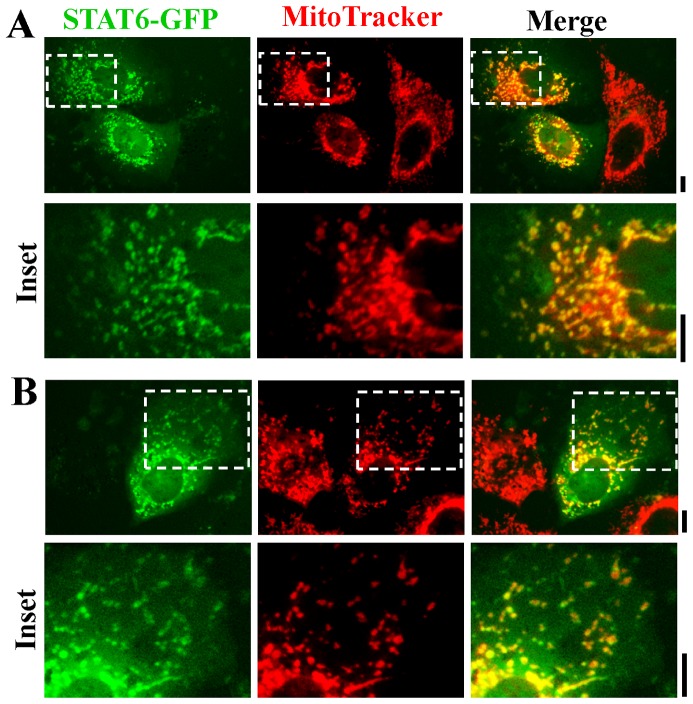
Targeting of STAT6-GFP to mitochondria in Hep3B cells assayed in live-cells using MitoTracker as the mitochondrial vital stain. Panels A and B. Hep3B cells in 35–mm plates were transfected with the STAT6-GFP expression plasmid and 2 days later the cultures were exposed to MitoTracker Red CMXRos at 100 nM for 15 min. After washing with PBS the live cells were imaged using a 40x water-immersion objective in green (STAT6-GFP) and red (MitoTracker). Insets are shown at high magnification within each panel. Scale bars = 10 µm. Quantitative colocalization analyses using the Pearson’s and Costes’ plugins in Image J confirmed colocalization between STAT6-GFP and MitoTracker fluorescence at a setting of *P*<0.05 in Panels A and B.

**Figure 7:**
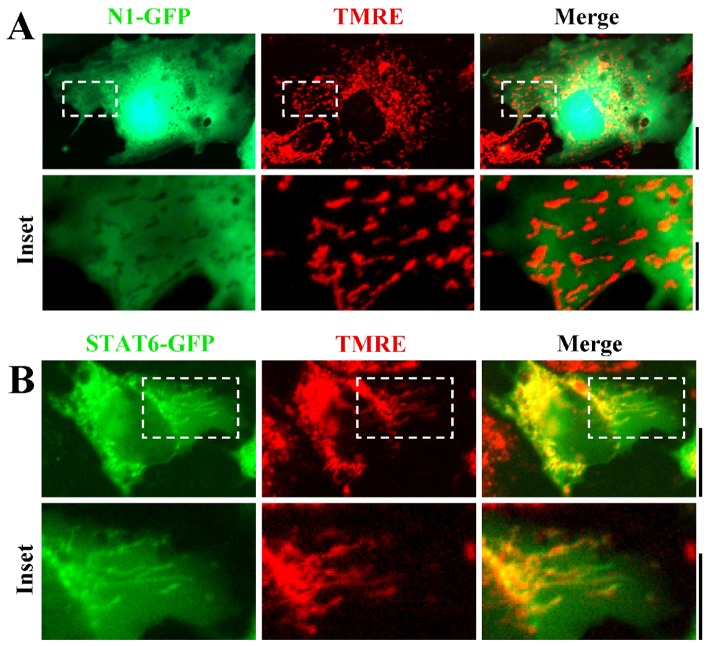
Targeting of STAT6-GFP but not of N1-GFP to mitochondria in Hep3B cells assayed in live-cells using TMRE as the mitochondrial vital stain. Hep3B cells in 35–mm plates were transfected with expression plasmids for N1-GFP (Panel A) or STAT6-GFP (Panel B) and 2 days later the cultures were exposed to TMRE at 5 nM for 15 min. After washing with PBS the live cells were imaged using a 40x water-immersion objective by two-color fluorescence in green (N1-GFP or STAT6-GFP) and red (TMRE). Insets are shown at high magnification within each panel. Scale bars = 10 µm except in Panel B, insets = 5 µm.

**Figure 8:**
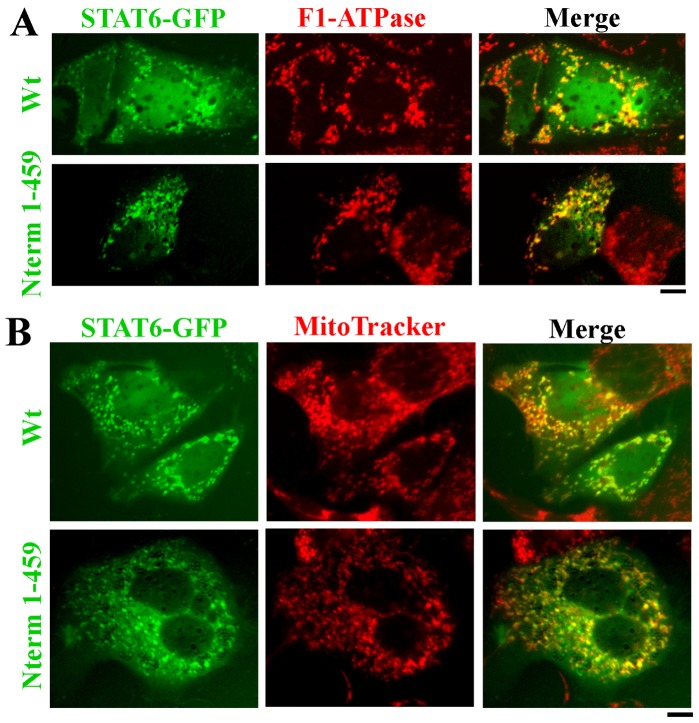
Targeting of STAT6^1–459^-GFP to mitochondria in Hep3B cells. Panels A and B. Hep3B cells in 35–mm plates were transfected with expression constructs for either the full-length or the 1-459 truncated version of STAT6-GFP. Two days later the plates were either fixed and immunostained for F1-ATPase (Panel A) or exposed to MitoTracker Red CMXRos at 100 nM for 15 min Panel B) and imaged as indicated in Materials and Methods. Scale bars = 10 µm.

The possibility that STAT6 might constitutively associate with mitochondria was investigated further in live-cell assays using STAT6-GFP imaging and marking mitochondria with either MitoTracker or tetramethylrhodamine ethyl ester (TMRE). Hep3B cells transfected with an exogenous vector expressing STAT6-GFP were imaged 2 days later together with labeling with vital labeling with MitoTracker (Fig. 6). The data show clear association of STAT6-GFP with MitoTracker-positive organelles in the cytoplasm (Fig. 6). This association was further explored by comparing the localization of STAT6-GFP to that of the empty N1-GFP protein, and using a different vital stain for mitochondria. Fig. 7A shows that N1-GFP did not localize with TMRE-positive mitochondria, while Fig. 7B shows that STAT6-GFP did. Fig. 8A, top row confirms this association of STAT6-GFP with mitochondria in cells that had expressed the GFP-tagged protein, and were then fixed and assayed for F1-ATPase immunofluorescence.

Using MitoProt II and other search algorithms, STAT6 did not have any obvious mitochondrial targeting sequence. The sequence dependence of this mitochondrial association was investigated by constructing GFP-tagged proteins representing the N-terminal half (amino acids 1–459) or the C-terminal half (amino acids 460–847). While the C-terminal half did not show any successful expression, the N-terminal half showed expression and clear mitochondrial localization, including in live-cell assays (Fig. 8A and B). It is noteworthy that this N-terminal STAT6-GFP truncation lacked the SH2 domain and the cytokine-activated Y641 phosphorylation site.

### Mitochondria-associated STAT6 peptides in MEFs from *STAT6^SH2-/SH2-^* mice

**Figure 9:**
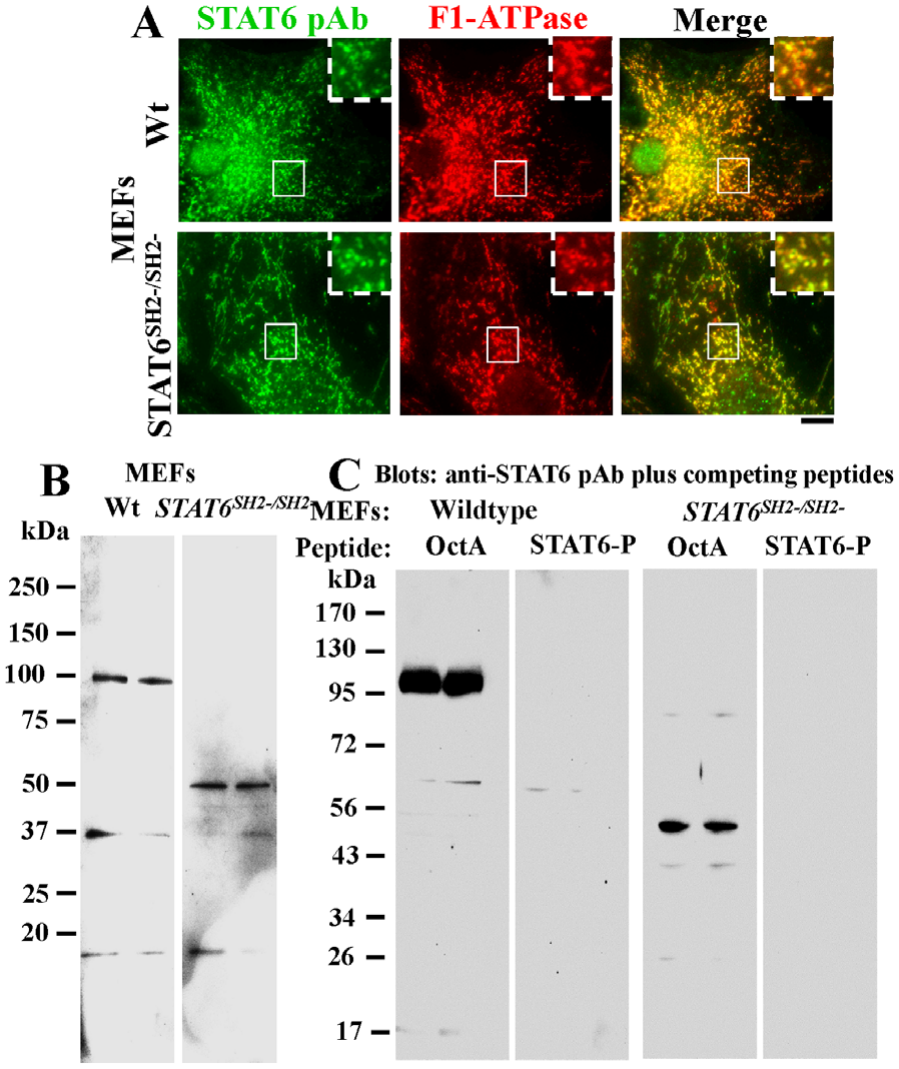
STAT6 associated with mitochondria in MEFs derived from wildtype (wt) or *STAT6^SH2-/SH2-^* mice. Panel A. MEFs derived from wt and *STAT6^SH2-/SH2-^* grown in 35–mm plates were washed using the digitonin-sucrose buffer and then immunostained for STAT6 and F1-ATPase. The cells were imaged using a 100x oil immersion objective. Insets represent the boxed regions within each frame. Scale bar = 10 µm. Panels B and C. Whole-cell extracts of MEFs derived from wt or *STAT6^SH2-/SH2-^*mice were evaluated (each in duplicate lane per variable) using Western blotting without (Panel B), and with peptide-competition (Panel C).

In seeking further insight into the association of STAT6 with mitochondria and the requirement or lack thereof of the SH2 domain, we turned to an investigation of MEFs derived from wild-type (wt) or the *STAT6^SH2-/-SH2-^* mice. The latter mice carrying deletions in the SH2 domain of STAT6 had been developed by Kaplan et al in 1996 [13] and have been widely distributed by The Jackson Labs and extensively used by investigators as STAT6-null mice in that belief that spleen and lymphoid cells from such mice “do not express STAT6 protein” i.e. the full-length STAT6 protein [13], [14], [15], [16]. In our hands, consistent with the STAT6^1–459^-GFP data (Fig. 8), MEFs from both wt and *STAT6^SH2-/-SH2-^* mice showed colocalization of anti-STAT6 pAb immunofluorescence with mitochondria (Fig. 9A). This was immune-specific in that the relevant STAT6 peptide blocked this immunofluoresence, even in MEFs from *STAT6^SH2-/-SH2-^* mice (not shown). Evaluation of STAT6-specific peptides in the wt and *STAT6^SH2-/-SH2-^* MEFs by Western blotting disclosed that while the wt contained the full-length protein (∼100 kDa) reactive with the anti-STAT6 pAb, *STAT6^SH2-/-SH2-^* MEFs expressed a ∼50 kDa STAT6-specific peptide (Fig. 9B). Importantly, both were competed for by the relevant STAT6 peptide but not the irrelevant OctA peptide (Fig. 8C). Moreover, the ∼50 kDa peptide was present in the mitochondria-enriched 7.5K pellet fraction isolated from *STAT6^SH2-/-SH2-^* MEFs which was verified to be immune-specific by peptide competition (Fig. 5A, rightmost lane in each blot section). Indeed, STAT6 peptides of molecular mass in the 40–50 kDa range were enriched in mitochondria-enriched sedimentation fractions of the 7.5K pellet derived from Hep3B cells (Fig. 5C). Taken together, the data in Figures 5, 8 and 9 indicate that the SH2 domain is not required for the association of STAT6 with mitochondria.

## Discussion

The present studies provide definitive evidence for the association of a STAT-protein family member with mitochondria. The previous inference by several investigators of an association between STAT3 and mitochondria has remained controversial primarily because of a lack of convincing imaging data placing STAT3 in or with mitochondria [1], [2], [3], [4]. Despite investigating different experimental conditions and preparative methods we failed to observe GFP-, DsRed- or Flag-tagged STAT3 in association with mitochondria. In contrast, the association of STAT6-GFP with mitochondria was readily observed in live-cell imaging assays. A GFP-tagged N-terminal fragment of STAT6 (1–459 amino acids) which lacked the SH2 domain and the cytokine-activated Y641 site associated with mitochondria suggesting that this association was constitutive and did not require cytokine-activated Tyr-phosphorylation. Moreover, we observed a ∼50 kDa STAT6 peptide in MEFs derived from the *STAT6^SH2-/SH2-^* widely distributed by a commercial vendor and extensively used by investigators since 1996 as a "STAT6-null mouse" in numerous studies [13], [14], [15], [16]. Grusby and colleagues had engineered this STAT6 knockout mouse by deleting the SH2 domain (amino acids 505–584)[13]. In our hands, MEFs derived from such mice showed STAT6-immunolocalization with mitochondria. This is consistent with our observation that an exogenously expressed and GFP-tagged N-terminal fragment (1–459) of STAT6 which lacked the SH2 domain (and the Y641 phosphorylation site) also associated with mitochondria.

In previous imaging studies of STAT6-GFP Chen and Reich [17] commented in 2010 on the punctate cytoplasmic appearance of STAT6-GFP, especially in COS1 cells transfected with importin β1 siRNA to inhibit trafficking to the nucleus (Fig. 7B in ref. [17]). Moreover, data from these authors show STAT6-GFP constitutively present in puncta in the cytoplasm (Fig. 2, top row in ref. [17]). However, these investigators did not consider the mitochondrial localization of STAT6-GFP.Indeed, in 2011 when the Reich group reported on their failure to observe STAT3-GFP in association with MitoTracker-positive cytoplasmic organelles [11], they did not appear to have investigated STAT6-GFP for this colocalization in the later study. In contrast to data reported in 2010 by the Reich group [17] that cells with a knockdown of importin-β1 (using siRNA) showed marked punctate accumulation of STAT6-GFP in the cytoplasm (we presume in mitochondria)(see Fig. 7 in ref. [17]), the data reported in 2011 by the same group show that STAT3-GFP did not behave in this manner upon importin-β1 knockdown (see Fig. 7 in ref. [11]). Thus, the juxtaposition of data from the two publications from 2010 and 2011 [11], [17] shows a clear difference in the cytoplasmic organellar targeting of STAT6-GFP and STAT3-GFP. Our present study suggests that the STAT6-GFP-positive cytoplasmic organelles observed by Chen and Reich [17] were likely to be mitochondria.

That proteins with major functions in the nucleus can also serve significant functions in the mitochondria is an emergent theme in cell biology. A recent example is the discovery that the nuclear telomerase complex protein TIN2 is imported into mitochondria, there processed by proteolytic cleavage to serve functions that include generation of reactive oxygen species [18]. That the full-length ∼100 kDa STAT6 undergoes specific proteolytic processing to a ∼65 kDa fragment has alkready been documented [19]. More recently, Chen et al [20] reported the recruitment of STAT6 to the endoplasmic reticulum in response to virus infection to serve antiviral functions independent of the Janus-activated kinases. We recently reported the constitutive association of STAT5a (and its GFP-tagged version) with the Golgi apparatus and the endoplasmic reticulum for the purposes of nongenomic functions in those organelles [21], [22]. Specifically, the acute knockdown of STAT5a/b using siRNAs led to a cystic dilatation of the endoplasmic reticulum and fragmentation of the Golgi apparatus and mitochondria even in enucleated cytoplasts of endothelial cells [21], [22]. Thus, more broadly, the association of STAT-family members with cytoplasmic organelles for nongenomic functions outside the nucleus is an exciting new area of research. Indeed, in the present study we have been able to definitively associate STAT6-GFP with mitochondria.

However, inferences of the organellar association of STAT proteins and their potential nongenomic functions in the cytoplasm need careful data evaluation. Thus a recent claim of cytokine-stimulated translocation of STAT5 (including of Tyr-phosphorylated STAT5) to mitochondria in leukemic T cells exposed to IL-3 rests on inadequately controlled cell-fractionation and immunofluorescence evidence [23] in that the data do not exclude the association of PY-STAT5 with cytoplasmic endosomes, as is already known for IL-6-activated PY-STAT3 [7], [8], [10]. There is a similar technical limitation in the cell fractionation data of Wegrzyn et al [1] and Jeong et al [3] for membrane-associated cytoplasmic STAT3 in the cytoplasm. In contrast, the present data, especially the live-cell STAT6-GFP imaging data, unequivocally establish the association of STAT6 with mitochondria.

STAT6 lacks an obvious mitochondrial import signal sequence. Nevertheless, the N-terminal 459 amino acids appear sufficient to mediate this mitochondrial association. That this mitochondrially-targeted fragment of STAT6 lacked the SH2 domain (amino acids 1–459) and the Y641 phosphorylation site indicates that the mitochondrial import mechanisms involved is likely independent of cytokine-mediated activation of STAT6. Although the immunogold EM localization studies (Fig. 4C) show STAT6 localization at the inner mitochondrial membrane and mitochondrial matrix, detailed submitochondrial targeting of STAT6-GFP and its function in that organelle await future studies.

To summarize, the present imaging studies of the cytoplasmic organellar localization of STAT6-GFP provide the first definitive evidence of the association of any STAT-protein family member with mitochondria.

## Materials and Methods

### Ethics Approval

The derivation of mouse embryo fibroblast (MEF) cultures using wild-type C57Bl/6J mice and breeder pairs of homozygous B6.129S2(C)-Stat6^tm1Gru^/J (Stock Number 005977) mice [13] obtained from The Jackson Labs, Bar Harbor, ME was carried out per a protocol approved by the New York Medical College Institutional Animal Care and Use Committee (IACUC).

### Antibodies and competing peptides

Rabbit polyclonal antibodies to STAT6 (sc-621x), glucose-6-phosphate dehydrogenase (sc-67165), the Flag tag (sc-807) and the respective relevant competing peptide to STAT6 (sc-621P) and the irrelevant OctA peptide (sc-835P) were obtained from Santa Cruz Biotechnology Inc. (Santa Cruz, CA). The anti-STAT6 antibody is reported by the manufacturer to be “an affinity purified rabbit polyclonal antibody raised against a peptide mapping at the C-terminus of STAT6 of human origin.” Murine mAbs to F1-ATPase (sc-58618) and COX IV (sc-58348) were also obtained from Santa Cruz. Rabbit pAb to COX IV (#4844) was obtained from Cell Signaling (Danvers, MA), while murine mAb to LAMP1 (#611042) was obtained from BD Biosciences-Pharmingen (San Diego, CA) while an mAb to STAT6 (S25420; designated “anti-IL-4 STAT”) was from Transduction Laboratories, Lexington, KY. Respective AlexaFluor 488- and AlexaFluor 594-tagged secondary donkey antibodies to rabbit, mouse or goat IgG were from Invitrogen Molecular Probes (Eugene, OR).

### Cell cultures

Human hepatoma Hep3B cells were obtained from the American Type Culture Collection (Camden, NJ) and grown in 6-well plates, 90 mm plates or T-75 flasks in Dulbecco’s Modification of Eagle’s medium (DMEM) supplemented with 10% fetal bovine serum (Atlanta Biologicals, Lawrenceville, GA), glucose (4.5 gm/lit), L-glutamine (4 mM) and sodium pyruvate (1 mM)(Mediatech, Inc., Manassas, VA)[5], [6], [7], [8]. Primary human pulmonary arterial endothelial (HPAEC) and smooth muscle (HPASMC) cells were purchased from Clonetics (San Diego, CA). Both were seeded into T-25, T-75 or 6-well plates coated with fibronectin, collagen and bovine serum albumin (respectively 1 µg/ml, 30 µg/ml and 10 µg/ml in coating medium), grown in media recommended by the vendor and used as previously reported [21]. As indicated in respective experiments, Hep3B cultures were exposed to recombinant human IL-6 for 30 min (10 ng/ml; R&D Systems, Minneapolis, MN). IL-6 was used as the activating cytokine in the present experiments using Hep3B cells because it is well established to activate acute phase plasma protein synthesis in these cells through activation of the STAT3 signaling pathway (see [5], [6], [7], [8] and citations therein).

### Plasmids and cell transfections

Expression plasmids for STAT3-EGFP, control N1-EGFP, STAT3-DsRed and STAT3-Flag were used as reported earlier [8]. STAT6-GFP expression plasmid was purchased from OriGene, Rockville, MD. The STAT6-GFP plasmid was further engineered to give the 1–459 truncated construct lacking the SH2 domain and the Y641 Tyr-phosphorylation site (STAT6^1–459^-GFP). The construct was verified by nucleotide sequencing. Expression vectors (usually 1-2 μg DNA per well in 6-well cultures) were transfected using PolyFect transfection reagent (Qiagen Inc, Valencia, CA) one-three days prior to experimental use. Live-cell imaging included vital staining for mitochondria using MitoTracker Red CMXRos (#M-7512; Molecular Probes, Eugene, Oregon) at 100 nM for 15 min in serum-free DMEM or using tetramethylrhodamine ethyl ester perchlorate (TMRE, Invitrogen, Carlsbad, CA) at 5 nM for 15 min in serum-free DMEM.

### Wild-type and *STAT6^SH2-/-SH2-^* mice and MEFs derived therefrom

Wild-type C57Bl/6J mice and breeder pairs of homozygous B6.129S2(C)-Stat6^tm1Gru^/J (Stock Number 005977) mice [13] were obtained from The Jackson Labs, Bar Harbor, ME and used to derive MEFs from 13 or 14-day-old embryos. The latter mice had been engineered in 1996 by Kaplan et al to lack the SH2 domain (amino acids 505–584) and spleen and thymus extracts reported to lack the full-length STAT6 protein by Western blotting (see Fig. 1 in ref. [13]). This mouse strain has been used extensively by other investigators [14], [15], [16](and additional citations available from the vendor’s website). Mice were bred without any experimental manipulation and female mice were euthanized using carbon dioxide when 13–14 days pregnant.

### Immunofluorescence microscopy of cells in culture

This was carried out typically in cultures in 6-well plates or in 35–mm dishes as described previously [7], [9], [21], [22]. Respective cultures were washed once with ice-cold PBS and then fixed using 4% cold paraformaldehyde in PBS (1 hr at 4°C) followed by washing 4 times with cold PBS. Alternatively, cultures were first washed 4 times (0.8–1 ml per well) using a digitonin/sucrose buffer [digitonin 50 µg/ml (Sigma, St. Louis, MO) in 0.3 M sucrose in ELB; 1 minute incubation on ice with intermittent rocking per wash] followed by paraformaldehyde fixation. The cultures were permeabilized by exposure to 0.1% Triton X-100 in PBS at room temperature for 8 min with gentle rocking, blocked for 2 hr using 2% (v/v) normal donkey serum (NDS, Jackson ImmunoResearch Labs, Inc., West Grove, PA) in PBS at room temperature (0.5 ml/well), and then incubated overnight with the respective primary antibodies (0.5 ml/well of antibody dilutions in the range 1∶100 to 1∶2000 as appropriate) in PBS containing 0.2% NDS with gentle rocking at 4°C. Subsequently the cultures were washed with PBS 4 times (2 ml/well each) with gentle rocking at room temperature for 3–5 min each wash, exposed to the respective AlexaFluor-tagged goat secondary antibodies (typically diluted to 1∶400 in PBS with 0.2% NDS) for 90–120 min (0.5 ml/well) at room temperature followed by 4 washes with PBS. When a second primary antibody to a different antigen on the same culture was investigated the process was repeated in its entirety beginning with reblocking. In sequential staining, care was taken to ensure that the different primary antibodies used were derived from different species (rabbit, mouse or goat) followed by different AlexaFluor-tagged donkey-derived secondary Abs. Cultures were finally counterstained with 4',6-diamidino-2-phenylindole (DAPI) to mark nuclei. For peptide competition assays, 1–5 µl of the respective antibody stock was mixed with 20–25 µl of the competing peptide stock (both as provided by the manufacturer), incubated at room temperature for 30 min, and then diluted to 0.5 or 1 ml in PBS containing 0.2% NDS prior to use in immunofluoresence assays as above or for use as probes in Western blotting assays (below).

Images were collected using a Zeiss AxioImager M2 motorized microscopy system with Zeiss W N-Achroplan 40X/NA0.75 and Zeiss EC Plan-Neofluor 100X/NA1.3 oil objectives equipped with an high-resolution RGB HRc AxioCam camera (1,388 × 1,040 pixel high-speed color capture mode) and AxioVision 4.8.1 software. Controls included secondary antibodies alone, peptide competition assays and multiple different antibodies towards the same antigen [21], [22]. All data within each experiment were collected at identical imaging settings; relevant sets of images were adjusted only for brightness/contrast and none were deconvolved.

### Quantitative colocalization image analyses

Dual channel colocalization analysis was performed on sections of the images representing cytoplasmic signals using the NIH Image J software and the Colocalization Test plugin (available as free downloads from www.macbiophotonics.ca/imagej/) with *P* set at <0.05. The Colocalization Test tool was uses the Costes image randomization algorithm [24], [25] to generate a correlation between pixels of two different colors (expressed as Pearson’s *R*). For each comparison, 1000 such random images were generated and the values of the random *R* [*R*(rand)] were compared to the actual *R*. The cutoff for the significance of colocalization, *P*-value, was set at <0.05 [24], [25]. 

### Immunogold electron microscopy

Thin-section immunogold EM was carried out by the Microscopy Core of the Office of Collaborative Science at New York University-Langone Medical Center [26]. Briefly, HPAEC or Hep3B in 35 mm cultures were washed with the digitonin-sucrose buffer and then fixed using 4% paraformaldehyde for 1 hr at 4°C. The cells were then scraped, collected as a pellet in an Eppendorf tube and embedded in situ and prepared for cryo-thin sectioning. The sections were then immunoprobed using anti-STAT6 pAb, unrelated pAb or no primary antibody and the immunoreactivity visualized using either Protein A- 15 nm gold beads or anti-rabbit-IgG-18 nm gold beads [26].

### Cell fractionation

Fractionation of Hep3B cells was carried out as previously described using hypotonic cell breakage using erythrocyte-lysis buffer (ELB)[5], [6], [7]. The postnuclear supernatant was adjusted to 0.25 M sucrose and was centrifuged at 7,500 *g* for 10 min (i.e. at 9000 rpm using a Sorvall SS-34 rotor) to obtain a mitochondria-enriched pellet fraction which was resuspended in 5 ml of a 0.25 M sucrose buffer (0.25 M sucrose in ELB) and resedimented twice. The 2x washed P7.5 pellet was further sedimented through a Percoll (30% v/v)-sucrose (0.25 M) gradient in an SW50.1 rotor (Beckman) at 22,000 rpm for 40 min. Gradient fractions were typically collected using a Pharmacia peristaltic pump from the bottom of the gradient, diluted using 0.25M sucrose/ELB, resedimented for 15 min at 15,000 *g* and the pellet resuspended usually in 100 µl ELB for subsequent Western blotting.

### Anti-TOM22-mAb magnetic bead column separation

The 2x washed P7.5K pellets were further fractionated into a mitochondria-enriched and a mitochondria–depleted fraction using the paramagnetic anti-TOM22–mAb bead separation kit (Miltenyi Biotec, Auburn, CA) with the immunoisolation of intact mitochondria carried out as per the manufacturer’s protocol.

### Protein quantitation and Western blotting

Protein concentrations of various cell fractions were determined using the BioRad Protein Assay (BioRad, Hercules, CA) and a bovine serum albumin standard (BSA, 2.0 mg/ml; Pierce, Rockford, IL). Western blotting was carried using electrophoresis through 4–20% polyacrylamide Criterion Precast Gels (1.0 mm; 18 well; BioRad) under reducing denaturing conditions, transfer to PVDF membrane (BioRad) and immunoimaging using the ECL detection kit (Amersham Biosciences, Inc, UK) or SuperSignal West chemiluminescent kit (Thermo Scientific, Rockford, IL) and BioMax Light film (Kodak, Inc., Rochester, NY). Immunoprobing for β-actin was used, when appropriate, for comparing cell extract loading between lanes.
